# Energy-Aware Computation Offloading of IoT Sensors in Cloudlet-Based Mobile Edge Computing

**DOI:** 10.3390/s18061945

**Published:** 2018-06-15

**Authors:** Xiao Ma, Chuang Lin, Han Zhang, Jianwei Liu

**Affiliations:** 1 Tsinghua National Laboratory for Information Science and Technology, Department of Computer Science and Technology, Tsinghua University, Beijing 100084, China; maxiao13@mails.tsinghua.edu.cn (X.M); chlin@tsinghua.edu.cn (C.L.); 2 School of Cyber Science and Technology, Beihang University, Beijing 100191, China; liujianwei@buaa.edu.cn

**Keywords:** mobile edge computing, QoS-aware, energy-aware, Internet of Things, heterogeneous wireless access

## Abstract

Mobile edge computing is proposed as a promising computing paradigm to relieve the excessive burden of data centers and mobile networks, which is induced by the rapid growth of Internet of Things (IoT). This work introduces the cloud-assisted multi-cloudlet framework to provision scalable services in cloudlet-based mobile edge computing. Due to the constrained computation resources of cloudlets and limited communication resources of wireless access points (APs), IoT sensors with identical computation offloading decisions interact with each other. To optimize the processing delay and energy consumption of computation tasks, theoretic analysis of the computation offloading decision problem of IoT sensors is presented in this paper. In more detail, the computation offloading decision problem of IoT sensors is formulated as a computation offloading game and the condition of Nash equilibrium is derived by introducing the tool of a potential game. By exploiting the finite improvement property of the game, the Computation Offloading Decision (COD) algorithm is designed to provide decentralized computation offloading strategies for IoT sensors. Simulation results demonstrate that the COD algorithm can significantly reduce the system cost compared with the random-selection algorithm and the cloud-first algorithm. Furthermore, the COD algorithm can scale well with increasing IoT sensors.

## 1. Introduction

With the rapid emergence and sharp growth of Internet of Things (IoT), enormous computation and communication requirements are induced, exceeding the capacity of current data centers and mobile networks [1,2,3,4]. Mobile edge computing, e.g., cloudlet-based mobile edge computing [5] and fog computing [6,7], introduces promising solutions to overcoming this challenge in a distributed manner. In mobile edge computing, ubiquitous communication and computation resource provisioning is enabled by shifting computation resources to the edge of the Internet, i.e., wireless access networks [8,9,10,11]. Due to the advantages of low latency and high bandwidth, many efforts have been devoted to exploring the potentials of mobile edge computing in practical applications, such as IoT  [12] caching [13,14,15], security services [16] and vehicular networks [17]. However, the resources of mobile edge computing are limited, especially in the scenario with dense population. To deal with this problem, cloud-edge interoperation frameworks have been proposed to provision scalable services [18,19].

**Figure 1:**
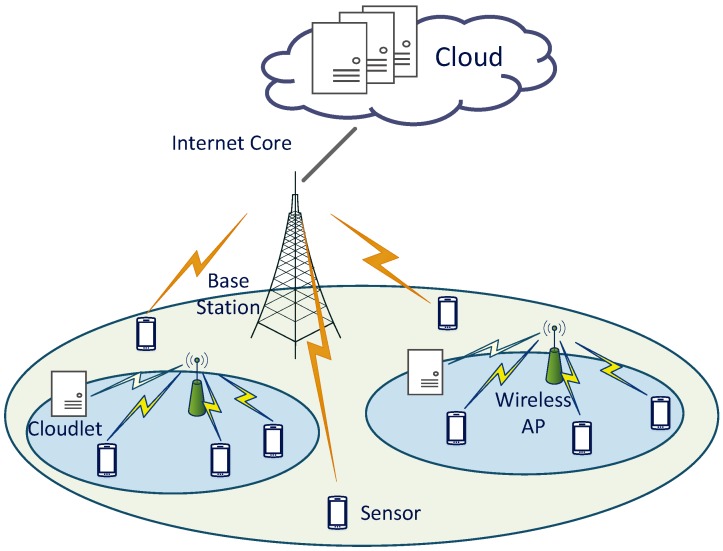
Cloud-assisted cloudlet-based mobile edge computing

In this paper, the cloud-assisted multi-cloudlet framework is introduced, as shown in Figure 1. A cloudlet is a multi-core computer or a cluster of well-connected computers [5]. Each sensor is able to offload its computation task to a cloudlet via a wireless AP, or to the remote cloud through the base station and Internet core. Due to the constrained computation resources of cloudlets and limited communication resources of wireless APs, the computation offloading decision of one sensor can have a significant influence on the processing delay and energy consumption of other sensors with identical computation offloading decisions. As processing delay and energy consumption of computation tasks are critical quality of service (QoS) metrics of IoT sensors, coordinating the computation offloading decisions of different IoT sensors is of vital importance.

This paper investigates the computation offloading decision problem of IoT sensors in the cloud-assisted multi-cloudlet framework. Many existing works have focused on computation offloading problems, including computation offloading problems of mobile devices [20,21,22,23], mobile data traffic offloading problems [24,25] and service migration problems [26]. Solving the computation offloading problem of IoT sensors in the cloud-assisted multi-cloudlet framework is challenging. First, IoT sensors are owned by different users with diversified interests. As IoT sensors with identical computation offloading decisions interact with each other, how to coordinate the computation offloading decisions of different sensors is challenging. This work solves the computation offloading decision problem of IoT sensors based on game theory, which is widely adopted to analyze systems with multiple selfish individuals [27]. Another challenge is that the cloud-assisted multi-cloud framework has heterogeneous computation and communication resources. Cloud resources can be provided on demand while computation resources of cloudlets are constrained. Each time one sensor changes its computation offloading decision, the computation and communication resources obtained by this sensor are changed simultaneously. Furthermore, due to the heterogeneous mobile networks (i.e., wireless access network and cellular network) in this framework, IoT sensors can have distinct energy consumption models when making different computation offloading decisions.

In this work, the computation offloading decision problem of IoT sensors is analyzed based on game theory. In more detail, the computation offloading decision problem of IoT sensors is formulated as a computation offloading game. To analyze the property of this game, the tool of potential game is introduced. Based on the definition of potential game, the condition of Nash equilibrium is derived, and the Computation Offloading Decision (COD) algorithm is further designed by leveraging the finite improvement property of potential game. In the COD algorithm, the improvement range is optimized in each improvement iteration, such that the computation complexity of the algorithm is significantly reduced. We implement extensive simulations to evaluate the COD algorithm. Simulation results demonstrate that the COD algorithm can effectively reduce the system cost (defined as the average weighted sum of processing delay and energy consumption of all sensors), compared with the random-selection algorithm and the cloud-first algorithm. Furthermore, the COD algorithm can scale well with increasing IoT sensors.

This paper is organized as follows. Section 2 presents the system model and Section 3 provides problem formulation and analysis. Algorithm design is presented in Section 4 and simulation results are illustrated and analyzed in Section 5. Finally, this work is concluded in Section 6.

## 2. System Model

This section presents the system model. As shown in Figure 1, there are a set of Γ={1,2,…,N} IoT sensors, each of which has a computation-intensive delay-sensitive computation task. Consider *K* cloudlets, to which IoT sensors can offload the computation tasks through wireless access points (APs). Since processing delay and energy consumption are two important factors that influence the performance of IoT sensors, this work considers these two issues as the metrics of QoS. In the cloudlet-based mobile edge computing framework, computation resources of each cloudlet are limited. In addition, IoT sensors connected to the identical wireless APs interfere with each other. Therefore, the performance of one sensor can be affected by the other sensors with identical computation offloading decisions. As both the computation process and the communication process play a significant role in computation offloading of IoT sensors, analysis of these two processes is provided as follows.

### 2.1. Computation Process

In the cloud-assisted cloudlet-based mobile edge computing framework, each IoT sensor *n* has a computation task $M_{n}$, which is defined as

(1) $$M_{n}\overset{\Delta }{=}\left\{d_{n},u_{n}\right\},$$

where $d_{n}$ represents the data block transmitted during computation offloading and $u_{n}$ represents the computation requirements of the computation task. Denote by $s=(s_{1},s_{2},\ldots ,s_{N})$ the computation offloading decision profile of sensors. Considering *K* cloudlets, then $s_{n}=i$ (i∈{1,2,…,K}) when the computation task of sensor *n* is offloaded to cloudlet *i*, and $s_{n}=0$ if sensor *n* offloads the computation task to the cloud. Denote by $S_{n}$ the strategy set of sensor *n*.

As scalable computation resources can be provided by the cloud, when the computation task is offloaded to the cloud, the computation rate of each sensor can be guaranteed, denoted as $f_{n}^{c}$. Denote by $f^{e}$ the computation rate of each cloudlet and $v_{n}$ the ratio of computation resources that sensor *n* can obtain when offloading computation task to the cloudlet. Then, the computation rate of sensor *n* can be represented as

(2) $$r_{n}^{\mathrm{comp}}\left(s\right)=\left\{\begin{array}{c} f^{e}\frac{v_{n}}{\overset{N}{\underset{m\in \Gamma :s_{m}=s_{n}}{\sum }}v_{m}}\;\;\;\;\;s_{n}>0\\ f_{n}^{c}\;\;\;\;\;\;\;\;\;\;\;\;\;\;\;\;\;\;\;s_{n}=0. \end{array}\right.$$

Thus, the computation delay of sensor *n* is

(3) $$t_{n}^{\mathrm{comp}}\left(s\right)=\frac{u_{n}}{r_{n}^{\mathrm{comp}}\left(s\right)}.$$

### 2.2. Communication Process

As shown in Figure 1, each IoT sensor can offload computation tasks to a cloudlet via a wireless AP. Consider the IoT sensors that offload computation tasks to the identical cloudlet via the wireless AP. The spectrum of this wireless AP is shared among these sensors. In this paper, the CSMA access control protocol in [28] is adopted to distribute the spectrum among these sensors. In this access control protocol, sensors connected to the wireless AP compete for the channel to transmit the computation tasks. Once one sensor captures this channel, the computation task of this sensor is transmitted in the entirety of the channel. Thus, the expected transmission rate of an IoT sensor can be computed as

(4) $$R_{n}^{e}\left(s\right)=b_{n}\frac{w_{n}}{\underset{m\in \Gamma :s_{m}=s_{n}}{\sum }w_{m}},$$

where $b_{n}$ is the transmission rate that sensor *n* can achieve when capturing this channel, and $w_{n}$ represents the probability of obtaining the channel.

As computation and communication resources of cloudlets are constrained, when cloudlets have dense population, IoT sensors can connect to the Internet core via the base station and further offload computation tasks to the cloud. With the development of cellular network, especially the rapid emergence and deployment of 5G network, sufficient spectrum is provided and extreme low latency is guaranteed. Thus, interference between IoT sensors are not considered when transmitting via the base station. According to the above analysis, the communication delay of sensor *n* can be summarized as

(5) $$t_{n}^{\mathrm{comm}}\left(s\right)=\left\{\begin{array}{c} \frac{d_{n}}{R_{n}^{e}\left(s\right)},\;\;\;\;\;\;\;\;\;\;\;\;\;s_{n}>0,\\ \frac{d_{n}}{R_{n}^{\mathrm{BS}}}+t_{\mathrm{RTT}},\;\;\;\;\;s_{n}=0. \end{array}\right.$$

Here, $R_{n}^{\mathrm{BS}}$ represents the transmission rate of sensor *n* in the cellular network, and $t_{\mathrm{RTT}}$ is the round trip delay between the base station and the cloud.

### 2.3. Delay and Energy Model

Processing delay and energy consumption are two important QoS metrics of mobile applications. Processing delay consists of computation delay and communication delay, which is given as

(6) $$t_{n}\left(s\right)=t_{n}^{\mathrm{comp}}\left(s\right)+t_{n}^{\mathrm{comm}}\left(s\right).$$

During the computation offloading of IoT sensors, energy consumption only contains the energy consumed to offload computation tasks to cloudlets or to the cloud. When offloading computation tasks to cloudlets, IoT sensors transmit data blocks via wireless APs. This work adopts the energy model similar to the energy model in [29]. The energy consumption when offloading to cloudlets $e_{n}^{e}$ contains three parts:

- scanning energy $e_{n}^{s}$: energy consumption during scanning available APs,
- transmission energy $e_{n}^{t}$: energy consumption during data transmission,
- maintaining energy $e_{n}^{m}$: energy consumed to maintain interfaces on during computation offloading.

Scanning energy $e_{n}^{s}$ can be represented by a constant. Transmission energy $e_{n}^{t}$ is given by

(7) $$e_{n}^{t}=a_{0}d_{n},$$

where $a_{0}$ is the energy consumed to transfer one unit of data via wireless APs. Maintaining energy $e_{n}^{m}$ is computed as

(8) $$e_{n}^{m}\left(s\right)=p_{n}^{m}\cdot t_{n}^{\mathrm{comm}}\left(s\right).$$

Here, $p_{n}^{m}$ is the transmission power of sensor *n*. Therefore, the energy consumption when offloading to cloudlets ($s_{n}\in \left\{1,2,\ldots ,K\right\}$) is

(9) $$e_{n}\left(s\right)=e_{n}^{s}+a_{0}d_{n}+p_{n}^{m}\cdot t_{n}^{\mathrm{comm}}\left(s\right).$$

When offloading computation tasks to the cloud through the base station and Internet core, the energy consumption of IoT sensors during computation offloading contains includes tail energy $e_{n}^{\mathrm{tail}}$ as well. Thus, the energy consumption when offloading to the cloud ($s_{n}=0$) is

(10) $$e_{n}\left(s\right)=e_{n}^{s^{\prime }}+e_{n}^{\mathrm{tail}}+a_{1}d_{n}+p_{n}^{m^{\prime }}\cdot t_{n}^{\mathrm{comm}}\left(s\right),$$

where $e_{n}^{s^{\prime }}$ and $p_{n}^{m^{\prime }}$ are scanning energy and maintaining power, respectively, when offloading to the cloud, and $a_{1}$ is the energy consumed to transfer one unit of data via the base station.

## 3. Problem Formulation and Analysis

In the cloud-assisted multi-cloudlet framework, IoT sensors contend for the computation and communication resources of the cloudlets and wireless APs to meet the QoS requirements of IoT applications. Since IoT sensors are owned by different individuals that pursue diversified interests, decentralized computation offloading decisions should be made to mimic the selfish property of the sensor users. As systems consisting of multiple selfish individuals are usually analyzed based on game theory [27], this work formulates the computation offloading problem of multiple IoT sensors as a computation offloading game. The details are presented as follows.

### 3.1. Computation Offloading Game

In the cloud-assisted multi-cloudlet framework, the computation offloading problem of IoT sensors is formulated as a computation offloading game. The players are the IoT sensors that have computation tasks to offload. The strategy of each player *n* (n∈Γ) is the computation offloading decision $s_{n}$ ($s_{n}\in \left\{0,1,\ldots ,K\right\}$). Denote by $s_{-n}$ the computation offloading decisions of all IoT sensors except *n*, i.e.,

(11) $$s_{-n}\overset{\Delta }{=}\left\{s_{1},\ldots s_{n-1},s_{n+1},\ldots ,s_{N}\right\}.$$

For each IoT sensor, the processing delay and energy consumption of the computation task are important QoS metrics, thus the cost function of IoT sensor *n* is defined as

(12) $$C_{n}\left(s\right)=C_{n}\left(s_{n},s_{-n}\right)=t_{n}\left(s\right)+\lambda_{n}\cdot e_{n}\left(s\right).$$

Here, $\lambda_{n}$ represents the weight parameter of energy consumption over processing delay, which is assigned by sensor *n* based on its interest.

According to the results in Section 2, when sensor *n* offloads the computation task to a cloudlet via a wireless AP (i.e., $s_{n}\in \left\{1,2,\ldots ,K\right\}$), the cost function of sensor *n* can be represented as

(13) $$\begin{array}{cc} C_{n}\left(s\right) & =\left(\frac{u_{n}\overset{N}{\underset{m\in \Gamma :s_{m}=s_{n}}{\sum }}v_{m}}{f^{e}v_{n}}+\frac{d_{n}\underset{m\in \Gamma :s_{m}=s_{n}}{\sum }w_{m}}{b_{n}w_{n}}\right)+\lambda_{n}\cdot \left(e_{n}^{s}+a_{0}d_{n}+p_{n}^{m}\cdot \frac{d_{n}\underset{m\in \Gamma :s_{m}=s_{n}}{\sum }w_{m}}{b_{n}w_{n}}\right)\\ & =\frac{u_{n}}{f^{e}}\cdot \frac{\overset{N}{\underset{m\in \Gamma :s_{m}=s_{n}}{\sum }}v_{m}}{v_{n}}+\frac{d_{n}}{b_{n}}\left(1+\lambda_{n}p_{n}^{m}\right)\cdot \frac{\underset{m\in \Gamma :s_{m}=s_{n}}{\sum }w_{m}}{w_{n}}+\left(\lambda_{n}e_{n}^{s}+\lambda_{n}a_{0}d_{n}\right). \end{array}$$

When sensor *n* offloads the computation task to the cloud (i.e., $s_{n}=0$), the cost function is

(14) $$\begin{array}{c} C_{n}^{0}=\left(\frac{u_{n}}{f_{n}^{c}}+\frac{d_{n}}{R_{n}^{\mathrm{BS}}}+t_{\mathrm{RTT}}\right)+\lambda_{n}\cdot \left[e_{n}^{s^{\prime }}+e_{n}^{\mathrm{tail}}+a_{1}d_{n}+p_{n}^{m^{\prime }}\cdot \left(\frac{d_{n}}{R_{n}^{\mathrm{BS}}}+t_{\mathrm{RTT}}\right)\right]. \end{array}$$

### 3.2. Analysis

Based on game theory, a Nash equilibrium of a game is defined as in Definition 1 [27].

**Definition** **1.** *A strategy profile* $s^{*}=\{s_{1}^{*},s_{2}^{*},\ldots ,s_{n}^{*}\}$ *is a Nash equilibrium, if for any* n∈Γ *and any* $s_{n}\in \left\{0,1,\ldots ,K\right\}$ *, it is satisfied that*

(15) $$C_{n}\left(s_{n}^{*},s_{-n}\right)\leq C_{n}\left(s_{n},s_{-n}\right).$$

According to Definition 1, when the computation offloading game of IoT sensors reaches Nash equilibrium, the strategy of each player is the best computation offloading decision that minimizes its cost function, given the strategies of all the other players. Thus, no player has the motivation to violate the current computation offloading decision.

For the computation offloading game of IoT sensors, each IoT sensor chooses the computation offloading strategy to minimize its cost function selfishly. Since the sensors offloading to the identical cloudlet via the wireless AP contend for the computation and communication resources, when one sensor *n* changes its strategy from $s_{n}$ to $s_{n}^{\prime }$, the cost of sensor *m* that has the strategy of $s_{n}^{\prime }$ (i.e., $s_{m}=s_{n}^{\prime }$) is influenced. Denote by $s_{m}^{*}$ the current computation offloading decision of sensor *m* and define $\Delta_{m}$ as

(16) $$\Delta_{m}=\min_{s_{m}\neq s_{m}^{*}}c_{m}\left(s_{m},s_{-m}\right)-c_{m}\left(s_{m}^{*},s_{-m}\right).$$

Then, $\Delta_{m}$ represents the smallest difference between all the other strategies and the current strategy of sensor *m*. Denote by $m^{*}$ the sensor that has the smallest $\Delta_{m}$, i.e.,

(17) $$m^{*}=\underset{s_{m}=s_{n}^{\prime }}{\arg\min\Delta_{m}}.$$

When sensor *n* changes its strategy from $s_{n}$ to $s_{n}^{\prime }$, the costs of sensors that offload computation tasks to $s_{n}^{\prime }$ increase due to the reduced computation and communication resources allocated to these sensors. It is intuitive that sensor $m^{*}$ is most likely to change its current computation offloading strategy, and the strategy is only changed when new sensors with larger Δ choose to offload the computation tasks to $s_{m}$ (i.e., $s_{n}^{\prime }$). When sensor $m^{*}$ chooses a new computation offloading strategy  $s_{m}^{\prime }$, it may further motivate sensors with the computation offloading strategy of $s_{m}^{\prime }$ and smaller Δ to change the computation offloading strategies.

According to the above analysis, when one sensor changes its computation offloading strategy, a chain reaction of strategy changes can be incurred. In the following work, we further explore the properties of the computation offloading game and analyze the existence of Nash equilibrium.

## 4. Algorithm Design

This section first derives the condition of Nash equilibrium by introducing the concept of a potential game [30]. Then, the Computation Offloading Decision (COD) algorithm is designed by exploiting the property of the computation offloading game.

### 4.1. Condition of Nash Equilibrium

According to [30], a potential game is defined as follows:

**Definition** **2.** *The computation offloading game of IoT sensors is a potential game if there exists a function* Θ(s) *such that if for any n,* $s_{n},s_{n}^{\prime }\in S_{n}$ *and* $s_{-n}\in \underset{m\in \Gamma \backslash \{n\}}{\prod }S_{n}$ *, if*

(18) $$C_{n}\left(s_{n},s_{-n}\right)<C_{n}\left(s_{n}^{\prime },s_{-n}\right),$$

*there is*

(19) $$\Theta \left(s_{n},s_{-n}\right)<\Theta \left(s_{n}^{\prime },s_{-n}\right).$$

*The function* Θ(s) *is called potential function.*

It is indicated from Definition 2 that in a potential game, when any IoT sensor n∈Γ changes its computation offloading decision from $s_{n}^{\prime }$ to $s_{n}$ so as to reduce its cost function, the value of the potential function also decreases. When the potential game reaches a Nash equilibrium, the potential function can not be further reduced. It has been proved that any finite potential game has a Nash equilibrium [30]. Therefore, if we want to derive the condition of Nash equilibrium in the computation offloading game of IoT sensors, we simply need to investigate the condition of a potential game.

**Theorem** **1.** *The computation offloading game of IoT sensors is a potential game if for every sensor* n∈Γ *, there  is*

(20) $$\frac{w_{n}}{\overset{N}{\underset{m\in \Gamma :s_{m}=s_{n}}{\sum }}w_{m}}=\frac{v_{n}}{\overset{N}{\underset{m\in \Gamma :s_{m}=s_{n}}{\sum }}v_{m}}.$$

**Proof.**  Substitute Equation (20) into Equation (13), and it is derived that when sensor *n* offloads the computation task to a cloudlet (i.e., $s_{n}\in \left\{1,2,\ldots ,K\right\}$), the cost function is

(21) $$C_{n}\left(s\right)=\left[\frac{u_{n}}{f^{e}}+\frac{d_{n}}{b_{n}}\left(1+\lambda_{n}p_{n}^{m}\right)\right]\cdot \frac{\underset{m\in \Gamma :s_{m}=s_{n}}{\sum }w_{m}}{w_{n}}+\left(\lambda_{n}e_{n}^{s}+\lambda_{n}a_{0}d_{n}\right).$$

Define $\Omega_{n}$ as

(22) $$\Omega_{n}=w_{n}\left[C_{n}^{0}-\left(\lambda_{n}e_{n}^{s}+\lambda_{n}a_{0}d_{n}\right)\right]/\left[\frac{u_{n}}{f^{e}}+\frac{d_{n}}{b_{n}}\left(1+\lambda_{n}p_{n}^{m}\right)\right],$$

where $C_{n}^{0}$ is represented in Equation (14). Then, it can be proved that there exists a potential function Θ(s)

(23) $$\Theta \left(s\right)=\frac{1}{2}\overset{N}{\underset{n=1}{\sum }}\overset{N}{\underset{m=1}{\sum }}w_{n}w_{m}f\left(s_{m}=s_{n}\right)f\left(s_{n}>0\right)+\overset{N}{\underset{n=1}{\sum }}\Omega_{n}w_{n}f\left(s_{n}=0\right)$$

in two cases:

Case 1: if $s_{n}>0$, $s_{n}^{\prime }>0$, then

(24) $$C_{n}\left(s_{n},s_{-n}\right)-C_{n}\left(s_{n}^{\prime },s_{-n}\right)=\left[\frac{u_{n}}{f^{e}}+\frac{d_{n}}{b_{n}}\left(1+\lambda_{n}p_{n}^{m}\right)\right]\cdot \frac{\underset{m\in \Gamma :s_{m}=s_{n}}{\sum }w_{m}-\underset{l\in \Gamma :s_{m}=s_{n}^{{}^{\prime }}}{\sum },w_{l}}{w_{n}}$$

and

(25) $$\begin{array}{cc} \Theta_{n}\left(s_{n},s_{-n}\right)-\Theta_{n}\left(s_{n}^{\prime },s_{-n}\right) & =w_{n}\left(\underset{m\in \Gamma :s_{m}=s_{n}}{\sum }w_{m}-\underset{l\in \Gamma :s_{m}=s_{n}^{{}^{\prime }}}{\sum }w_{l}\right)\\ & =\frac{{\left(w_{n}\right)}^{2}}{\left[\frac{u_{n}}{f^{e}}+\frac{d_{n}}{b_{n}}\left(1+\lambda_{n}p_{n}^{m}\right)\right]}\left[C_{n}\left(s_{n},s_{-n}\right)-C_{n}\left(s_{n}^{\prime },s_{-n}\right)\right]. \end{array}$$

In this case, if $C_{n}\left(s_{n},s_{-n}\right)-C_{n}\left(s_{n}^{\prime },s_{-n}\right)>0$, it is satisfied that $\Theta_{n}\left(s_{n},s_{-n}\right)-\Theta_{n}\left(s_{n}^{\prime },s_{-n}\right)>0$.

Case 2: if $s_{n}>0$, $s_{n}^{\prime }=0$, then

(26) $$C_{n}\left(s_{n},s_{-n}\right)-C_{n}\left(s_{n}^{\prime },s_{-n}\right)=[\frac{u_{n}}{f^{e}}+\frac{d_{n}}{b_{n}}\left(1+\lambda_{n}p_{n}^{m}\right)]\cdot \frac{\underset{m\in \Gamma :s_{m}=s_{n}}{\sum }w_{m}}{w_{n}}+\left(\lambda_{n}e_{n}^{s}+\lambda_{n}a_{0}d_{n}\right)-C_{n}^{0},$$

and

(27) $$\begin{array}{cc} \Theta_{n}\left(s_{n},s_{-n}\right)-\Theta_{n}\left(s_{n}^{\prime },s_{-n}\right) & =w_{n}\left(\underset{m\in \Gamma :s_{m}=s_{n}}{\sum }w_{m}-\underset{l\in \Gamma :s_{m}=s_{n}^{{}^{\prime }}}{\sum }w_{l}\right)\\ & =\frac{{\left(w_{n}\right)}^{2}}{\left[\frac{u_{n}}{f^{e}}+\frac{d_{n}}{b_{n}}\left(1+\lambda_{n}p_{n}^{m}\right)\right]}\left[C_{n}\left(s_{n},s_{-n}\right)-C_{n}\left(s_{n}^{\prime },s_{-n}\right)\right]. \end{array}$$

In this case, if $C_{n}\left(s_{n},s_{-n}\right)-C_{n}\left(s_{n}^{\prime },s_{-n}\right)>0$, $\Theta_{n}\left(s_{n},s_{-n}\right)-\Theta_{n}\left(s_{n}^{{}^{\prime }},s_{-n}\right)>0$ is satisfied too.

Therefore, if the condition in Equation (20) is satisfied in the computation offloading game of IoT sensors, the game is a potential game with the potential function presented in Equation (23). ☐

### 4.2. Computation Offloading Decision Algorithm

It has been proved that the computation offloading game of IoT sensors is a potential game when the condition in Equation (20) is satisfied. In a potential game, the Nash equilibrium can always be achieved after finite improvement iterations, which is called finite improvement property [30]. This part provides the design of the Computation Offloading Decision (COD) algorithm by exploiting the finite improvement property of the computation offloading game. The details are shown in Algorithm 1.

Denote by $\rho_{n}^{t}$ the better strategy set of sensor *n* in the *t*th iteration: if $C_{n}\left(s_{n}^{t-1},s_{-n}\right)>C_{n}\left(k,s_{-n}\right)$, then $k\in \rho_{n}^{t}$. $d_{n}^{t}$ represents the improvement decision of sensor *n* in the *t*th iteration and $\xi_{n}^{t}$ is the improvement range of cost function defined as $\xi_{n}^{t}=C_{n}\left(s_{n}^{t-1},s_{-n}\right)-C_{n}\left(d_{n}^{t},s_{-n}\right)$.

**Algorithm 1** Computation Offloading Decision (COD) algorithm
**Input:**
   Computation tasks of sensors $M_{n}$(n∈Γ).
**Output:**
   Computation offloading strategy of sensors $s_{n}$(n∈Γ).   **Initialization**   Each sensor initially offloads its computation task to the cloud, $s_{n}^{0}=0$ (n∈Γ).
1:Compute the cost of each sensor *n* when offloading the computation task to the cloud, $C_{n}^{0}$, based on Equation (14).2:**for** each improvement iteration *t* **do**3: **for** each sensor n∈Ψ(t−1) **do**4:  obtain the computation and communication resources allocated to sensor *n* when offloading the computation task to each cloudlet according to $s_{-n}^{t-1}$.5:  compute the better strategy set $\rho_{n}^{t}$ based on Equations (13) and (14).6:  choose the improvement decision $d_{n}^{t}$ from $\rho_{n}^{t}$ to minimize the cost function.7: **end for**8: **if** the better strategy set of all sensors $\overset{N}{\underset{n=1}{⋃}}\rho_{n}^{t}\neq \emptyset$ **then**9:  each sensor *n* with $\rho_{n}^{t}\neq \emptyset$ competes for the improvement opportunity, and the sensor with larger $\xi_{n}^{t}$ is endowed with higher priority of winning the contention.10:  **if** sensor *n* wins the contention **then**11:   $s_{n}^{t}=d_{n}^{t}$.12:  **end if**13: **else**14:  **break**;15: **end if**16:
**end for**



It is indicated in Definition 2 that the potential function decreases when each player changes its strategy to reduce its cost function. In addition, the computation offloading game of IoT sensors has a finite improvement property. As in each improvement iteration sensors with larger $\xi_{n}^{t}$ are endowed with a higher priority of winning the improvement opportunity (as presented in step 9), the improvement range of the potential function is optimized in each iteration. Therefore, the number of improvement iterations is reduced and the COD algorithm can have good scalability.

## 5. Simulations and Results

In this section, extensive simulations are conducted to evaluate the COD algorithm. Consider the simulation scenario in which there are *N* IoT sensors, each of which has a computation task $M_{n}=\left\{d_{n},u_{n}\right\}$ to offload. The simulations take the face recognition application in [31] as the example of the IoT computation tasks. As IoT sensors have heterogeneous computation requests, the data load $d_{n}$ and computation requirements $u_{n}$ follow normal distribution in these simulations, with the parameters of (420, 42) KB and (2, 0.2) Giga CPU cycles, respectively. There are 10 cloudlets, each of which has the computation capacity of 24 GHz. IoT sensors can offload computation tasks to each cloudlet via a wireless AP. The transmission rates of these wireless APs follow normal distribution, with the expectation of 5 Mbps and the standard deviation of 1 Mbps. When offloading the computation task to the cloud, a T2.nano instance is provided to serve each sensor, the computation rate of which is 2.4 GHz [32]. The upload rate of each sensor to the base station is 2 Mbps. The round trip delay from the base station to the cloud is 50 ms [33,34]. The energy consumption parameters are based on the measurement results in [29], as shown in Table 1.

The performance of the COD algorithm is compared with the random-selection algorithm and the cloud-first algorithm. In the random-selection algorithm, each IoT sensor randomly selects a computation offloading decision among the cloudlets and the cloud. In the cloud-first algorithm, all IoT sensors offload the computation tasks to the cloud.

### 5.1. Performance of the COD Algorithm

**Figure 2:**
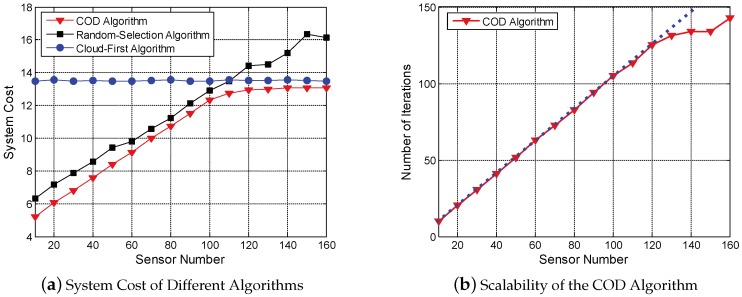
Performance of the COD Algorithm.

Defining the system cost as the average cost of all IoT sensors,

(28) $$C^{\mathrm{sys}}=\frac{\overset{N}{\underset{n=1}{\sum }}C_{n}\left(s\right)}{N},$$

then the system costs of the three algorithms are illustrated as Figure 2a.

As shown in Figure 2a, the COD algorithm can significantly reduce the system cost compared with the random-selection algorithm and the cloud first algorithm. In the cloud-assisted system, the base station provides abundant communication resources and the cloud has scalable computation resources. Thus, the system cost of the cloud-first algorithm remains almost unchanged with increasing sensor number.

When the sensor number is small, the cloudlets can provide sufficient computation resources and the wireless APs have abundant communication resources, offloading computation tasks to cloudlets yields lower costs than offloading to the cloud. In the COD algorithm and the random-selection algorithm, part of IoT sensors offload computation tasks to cloudlets, thus the system costs are lower than the cloud-first algorithm. As sensor number increases, the computation resources of cloudlets and the communication resources of wireless APs become scarce. The system costs of the COD algorithm and the random-selection algorithm increase rapidly with increasing sensor number. In the COD algorithm, the IoT sensor with the largest improvement range is endowed with the highest priority to update the computation offloading strategy in each iteration. Thus, the system cost of the COD algorithm increases slower than the random-selection algorithm, and finally approaches the result of the cloud-first algorithm.

To evaluate the scalability of the COD algorithm, simulations are further implemented to record the number of improvement iterations with different sensor numbers. The results are shown in Figure 2b. It is indicated that the number of iterations increases approximately linearly with the number of sensors. As revealed in Algorithm 1, the computation complexity of each improvement iteration is O(*NK*). Therefore, the computation complexity of the COD algorithm approaches O(*N*^2^*K*), and the COD algorithm scales well with increasing sensor number.

### 5.2. Influence of Different Parameters

**Figure 3:**
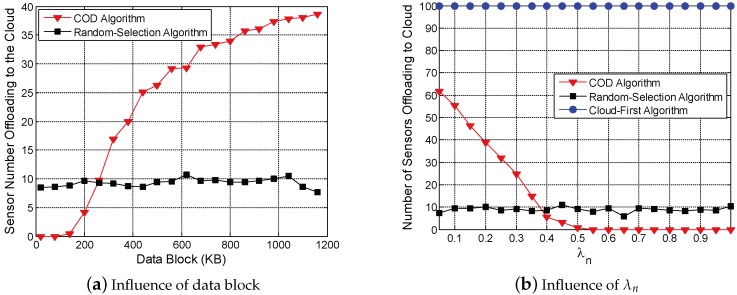
Influence of different parameters.

This part evaluates the influence of different parameters on the results of the COD algorithm. Simulations are conducted to record the number of sensors offloading to the cloud (the overall number of sensors *n* = 100). The results are illustrated as Figure 3a,b.

Figure 3a presents the influence of the size of trasmitted data block during computation offloading. In the random-selection algorithm, the number of sensors offloading to the cloud almost remains unchanged, since computation offloading strategies of sensors are selected randomly. As the communication resources of wireless APs are limited, the costs of sensors increase with growing data block when offloading the computation tasks to cloudlets. Therefore, increasing sensors offload the computation tasks to the cloud in the COD algorithm.

Figure 3b illustrates the influence of weight parameter of energy consumption over processing delay ($\lambda_{n}$). In this simulation, the transmitted data block is 420 KB, and the sensor number *n* = 100. According to [29], the energy consumption when transmitting the data block via the base station is higher than transmitting via wireless APs. As $\lambda_{n}$ increases, energy consumption occupies more in the cost of sensors. Thus, in the COD algorithm, the number of sensors offloading to the cloud (via the base station) decreases with increasing $\lambda_{n}$.

## 6. Conclusions

This work has investigated the computation offloading decision problem of IoT sensors in the cloud-assisted multi-cloudlet framework. Theoretic analysis has been presented and the condition of Nash equilibrium has been derived based on the potential game. By exploiting the finite property of the computation offloading game of IoT sensors, the COD algorithm has been proposed. Simulation results have demonstrated that the COD algorithm can effectively reduce the system cost compared with the random-selection algorithm and the cloud-first algorithm. In addition, the complexity of the COD algorithm is O(*N*^2^*K*), thus this algorithm can scale well with increasing IoT sensors.

## Figures and Tables

**Table 1:** Energy consumption parameters [29].

	Wireless AP	Base Station
Scanning Energy (J)	5.9	3.5
Transmission Power (J/KB)	0.007	0.025
Maintaining Power (W)	0.05	0.02
Tail Energy (J)	0	7.75
